# Three New Steroidal Glycosides from the Roots of *Cynanchum auriculatum*

**DOI:** 10.3390/molecules16021901

**Published:** 2011-02-23

**Authors:** Yu Lu, Hong-Li Teng, Guang-Zhong Yang, Zhi-Nan Mei

**Affiliations:** 1 Laboratory for Natural Product Chemistry, College of Pharmacy, South Central University for Nationalities, Wuhan 430074, China; 2 Guangxi Institute of Minority Medicine, Nanning 530001, China

**Keywords:** *Cynanchum auriculatum*, steroidal glycosides, cyanoauriculosides

## Abstract

Three new steroidal glycosides, cyanoauriculosides F, G and H (**1**-**3**), were isolated from the roots of *Cynanchum auriculatum* (Asclepiadaceae) along with two known steroidal derivatives. On the basis of spectroscopic analysis and chemical methods, their structures were identified as 20-*O*-acetyl-8,14-seco-penupogenin-8-one 3-*O*-*α*-L-cymaropyranosyl-(1→4)-*β*-D-cymaropyranosyl-(1→4)-*α*-L-diginopyranosyl-(1→4)-*β*-D-cymaropyranoside (**1**), 2′,3′-*Z*-gagaminine 3-*O*-*α*-L-cymaropyranosyl-(1→4)-*β*-D-cymaro-pyranosyl-(1→4)-*α*-L-diginopyranosyl-(1→4)-*β*-D-cymaropyranoside (**2**), 17-*O*-acetyl-kidjoranin 3-*O*-*α*-L-cymaropyranosyl-(1→4)-*β*-D-cymaropyranosyl-(1→4)-*α*-L-cymaro-pyranosyl-(1→4)-*β*-D-digitoxopyranosyl-(1→4)-*β*-D-digitoxopyranoside (**3**), gagaminine 3-*O*-*α*-L-cymaropyranosyl-(1→4)-*β*-D-cymaropyranosyl-(1→4)-*α*-L-digino-pyranosyl-(1→4)-*β*-D-cymaropyranoside (**4**) and wilfoside D1N (**5**).

## 1. Introduction

**Figure 1:**
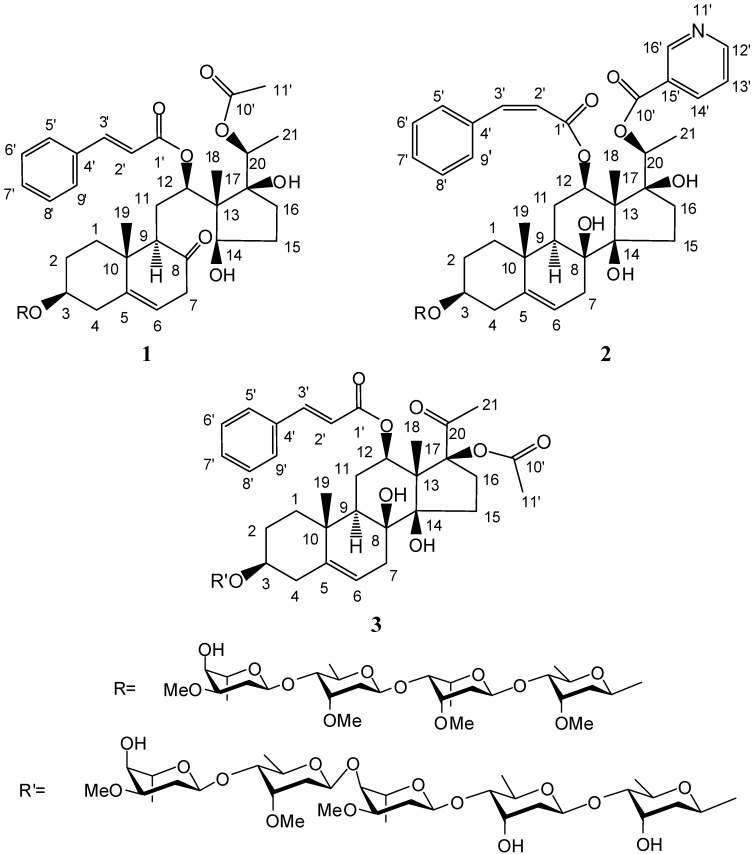
Structures of new compounds **1**–**3**.

*Cynanchum auriculatum* is a famous traditional medicine widely used in south China for the prevention of hair graying, strengthening sinews and bones, and enhancing immunity [1]. In previous papers, we reported the isolation of five new C-21 steroidal glycosides, named cyanoauriculosides A-E, from the roots of *C. auriculatum* [2]. Many C-21 steroidal glycosides isolated from *C. auriculatum* species have shown certain antitumor activities *in vitro* [3,4]. In a further phytochemical investigation of traditional Chinese medicinal plants to search for novel biologically active compounds, three new steroidal glycosides named cyanoauriculosides F-H (**1–3**, Figure 1) were obtained from the roots of *Cynanchum auriculatum* (Asclepiadaceae), along with two known steroidal derivatives. All the structures were established on the basis of spectroscopic analysis and chemical methods. 

## 2. Results and Discussion

Compound **1**, obtained as a white amorphous powder, showed a positive reaction in the *Libermann-Buchard* and *Keller-Killiani* tests, indicating the presence of a steroidal skeleton with a 2-deoxysugar moiety [5]. Its molecular formula C_60_H_90_O_20_ was deduced from the HRESIMS spectrum (*m/z* 1,165.5694 [M+Cl]^-^, calcd 1,165.5713). The ^1^H-NMR and ^13^C-NMR data (Table 1, Table 2) suggested that **1** was a C-21 steroidal glycoside. The ^1^H-NMR spectrum showed three Me groups at *δ*_H_ 2.02 (s), 1.57 (3H, d, *J* = 5.8 Hz) and 1.31 (s), three oxygenated CH groups at *δ*_H_ 5.17 (1H, m), 4.03 (1H, m) and 3.85 (1H, m), one olefinic proton at *δ*_H_ 5.26 (1H, br s), one cinnamoyl group at *δ*_H_ 6.81 (1H, d, *J* = 16.0 Hz), 8.02 (1H, d, *J* = 16.0 Hz), 7.65 (2H, m) and 7.33 (3H, m) which was supported by the ion fragment at *m/z* = 147 [C_9_H_7_O_2_]^+^ arising from the aglycone moiety. From ^1^H-NMR, ^13^C-NMR, HSQC and HMBC data, one acetyl group was also identified by the observation of a proton signal at *δ*_H_ 1.96 (3H, s) and two carbon signals at *δ*_C_ 170.5, 21.6. Comparison of the ^13^C-NMR data of the aglycone portion of **1** with that of penupogenin [6,7], showed that the major difference between the two substances was the presence of an additional acetyl group in **1**, and the fact that the chemical shift of C-20 in **1** was deshielded by *ca.* 6 ppm. This observation suggested that the extra acetyl group was located at C-20, which was supported by HMBC correlation between H-20 (*δ*_H_ 4.03, m) and C-10′ (*δ*_C_ 170.5). Additionally, the chemical shifts of **1** are different from those of penupogenin at C-7 (+8.1 ppm), C-9 (+13.2 ppm), C-14 (-7.4 ppm) and C-18 (+8.9 ppm), and the chemical shift of C-8 appears at *δ* 209.8. It could be further speculated that the hydroxy group at C-8 was oxidized into a carbonyl, which was supported by HMBC correlations between H-14 (*δ*_H_ 4.95, m) and C-18 (*δ*_C_ 20.3), and between H-9 (*δ*_H_ 2.21, m) and C-8 (*δ*_C_ 209.8). Thus, the aglycone of compound **1** was determined to be 20-*O*-acetyl-8,14-seco-penupogenin-8-one. In the NOESY spectrum, NOE correlations between H-9 (*δ*_H_ 2.21, m) and H-12 (*δ*_H_ 5.17, m) provided evidence for a *β*-linked 12-O-cinnamoyl group. Based on the literature [8], the stereochemistry of the C-14 hydroxyl group was assigned as *β*. 

With respect to the glycosidic portion, it contained four anomeric C-atoms with signals at *δ* (C) 96.5 (C1^I^), 101.0 (C1^II^), 99.5 (C1^III^) and 99.1 (C1^IV^), corresponding to anomeric H-atom signals at *δ* (H) 5.10 (overlap), 5.10 (overlap), 4.95 (overlap) and 5.05 (overlap), which indicated that there were four sugar moieties in **1**. Acidic hydrolysis of **1** afforded a sugar mixture of cymarose and diginose, identified by TLC comparison with authentic samples. Comparing the ^13^C-NMR spectrum with that of penupogenin showed that the chemical shift of **1** are different from those of penupogenin at C-2 (-2.3 ppm), C-3 (+5.4 ppm) and C-4 (-4.4 ppm), due to glycosidation, therefore the sugar moiety was linked to the C (3)-O of the aglycone. Furthermore, HMBC correlations between H-C (1^I^) at *δ* (H) 5.10 and C (3) at *δ* (C) 76.9 were observed. Signals of each sugar unit (Table 2) were assigned by the HSQC, HMBC and ^1^H-^1^H COSY analyses and sugar moieties were identified as two *β*-D-cymaropyranosyls, one *α*-L-diginopyranosyl and one *α*-L-cymaropyranosyl. The sequence of the sugar chain was determined by the HMBC spectrum, in which distinct correlations between H-C (1^I^) at *δ* (H) 5.10 and C (3) at *δ* (C) 76.9, between H-C (1^II^) at *δ* (H) 5.10 and C (4^I^) at *δ* (C) 82.4, between H-C (1^III^) at *δ* (H) 4.95 and C (4^II^) at *δ* (C) 74.6, and between H-C (1^IV^) at *δ* (H) 5.05 and C (4^III^) at *δ* (C) 82.4. Thus, compound **1** was determined to be 20-*O*-acetyl-8,14-seco-penupogenin-8-one 3-*O*-*α*-L-cymaro-pyranosyl-(1→4)-*β*-D-cymaropyranosyl-(1→4)-*α*-L-diginopyranosyl-(1→4)-*β*-D-cymaropyranoside, and named cyanoauriculoside F.

Compound **2**, obtained as a white amorphous powder, showed positive reactions in the *Libermann-Buchard* and *Keller-Killiani* tests, indicating again the presence of a steroidal skeleton with a 2-deoxysugar moiety [5]. Its molecular formula C_64_H_91_NO_20_ was deduced from the HRESIMS spectrum (*m/z* 1,216.6033 [M+Na]^+^, calcd 1,216.6032). The ^1^H-NMR and ^13^C-NMR data (Table 1, Table 2) suggested that **2** was a C-21 steroidal glycoside.

**Table 1:** NMR data of the aglycone moieties of compounds **1-3** *δ* in ppm, *J* in Hz. *

No	1		2		3	
	*δ*_C_	*δ*_H_	*δ*_C_	*δ*_H_	*δ*_C_	*δ*_H_
1	39.5	1.09 (m), 1.82 (m)	39.2	1.11 (m), 1.83 (m)	39.0	1.10 (m), 1.82 (m)
2	29.8	1.78 (m), 2.10 (m)	29.8	1.75 (m), 2.03 (m)	29.9	1.79 (m), 2.09 (m)
3	76.9	3.85 (m)	77.6	3.81 (m)	77.7	3.84 (m)
4	38.6	2.42 (m), 2.56 (m)	38.2	2.40 (m), 2.52 (m)	39.3	2.41 (m), 2.52 (m)
5	140.5		139.1		139.3	
6	118.9	5.26 (br s)	119.3	5.31 (br s)	119.2	5.30 (m)
7	41.8	2.84 (m), 3.31 (m)	33.6	2.30 (m), 2.45 (m)	34.7	2.32 (m), 2.48 (m)
8	209.8		74.1		74.4	
9	57.2	2.21 (m)	44.0	1.71 (m)	44.6	1.72 (m)
10	38.0		37.2		37.5	
11	25.4	2.18 (m), 2.30(m)	25.1	2.15 (m), 2.29 (m)	25.1	2.16 (m), 2.32 (m)
12	74.6	5.17 (m)	74.5	5.18 (m)	73.7	5.18 (m)
13	57.2		56.9		58.2	
14	81.5	4.95 (m)	88.8		89.6	
15	34.2	2.11 (m)	34.7	2.09 (m)	33.9	2.12 (m)
16	33.4	2.02 (m), 3.26 (m)	33.8	2.03 (m), 3.23 (m)	33.1	2.05 (m), 3.24 (m)
17	87.7		87.2		92.5	
18	20.3	2.02 (s)	11.0	2.02 (s)	10.8	2.04 (s)
19	18.9	1.31 (s)	18.0	1.33 (s)	18.3	1.32 (s)
20	76.8	4.03 (m)	76.3	3.93 (m)	210.2	
21	14.9	1.57 (d, *J* = 5.8)	15.3	1.54 (d, *J* = 6.0)	27.9	2.49 (s)
1′	167.8		166.6		166.0	
2′	119.4	6.81 (d, *J* = 16.0)	120.0	6.80 (d, *J* = 12.0)	119.4	6.82 (d, *J* = 16.0)
3′	145.7	8.02 (d, *J* = 16.0)	144.6	7.96 (d, *J* = 12.0)	145.1	7.99 (d, *J* = 16.0)
4′	135.0		134.2		135.1	
5′	128.8	7.65 (m)	128.1	7.62 (m)	128.7	7.64 (m)
6′	129.3	7.33 (m)	130.0	7.35 (m)	129.4	7.37 (m)
7′	130.7	7.33 (m)	129.0	7.35 (m)	130.8	7.37 (m)
8′	129.3	7.33 (m)	130.0	7.35 (m)	129.4	7.37 (m)
9′	128.8	7.65 (m)	128.1	7.62 (m)	128.7	7.64 (m)
10′	170.5		164.6		170.6	
11′	21.6	1.96 (s)			21.2	2.06 (s)
12′			151.6	9.59 (d, *J* = 1.6)		
13′			123.9	7.28 (d, *J* = 7.2)		
14′			137.4	8.40 (d, *J* = 7.8)		
15′			127.1			
16′			153.7	8.79 (dd, *J* = 5.6, 1.6)		

* Compound **1** and **3**: in C_5_D_5_N; compound **2**: in CD_3_OD.

**Table 2:** NMR data of the sugar moieties of compounds **1-3** *δ* in ppm, *J* in Hz. *

No	1		2		3	
	*δ*_C_	*δ*_H_	*δ*_C_	*δ*_H_	*δ*_C_	*δ*_H_
β-D-cym			β-D-cym		β-D-digit	
1^I^	96.5	5.10 (overlap)	96.0	5.23 (overlap)	96.4	4.89 (d, *J* = 9.5)
2^I^	35.2	1.76 (m), 2.37 (m)	35.2	1.73 (m), 2.30 (m)	39.2	1.79 (m), 2.06 (m)
3^I^	77.5	3.88 (m)	77.6	3.88 (m)	67.2	4.64 (m)
4^I^	82.4	3.44 (m)	82.3	3.46 (m)	83.5	3.51 (m)
5^I^	69.2	4.18 (m)	69.3	4.19 (m)	68.6	4.27 (m)
6^I^	18.8	1.49 (d, *J* = 6.1)	18.5	1.50 (m)	18.6	1.51 (d, *J* = 6.0)
-OMe	57.2	3.50 (s)	57.2	3.46 (s)		
α-L-digin			α-L-digin		β-D-digit	
1^II^	101.0	5.10 (overlap)	100.9	5.14 (overlap)	99.7	5.12 (d, *J* = 10.0)
2^II^	32.5	2.00 (m), 2.33 (m)	32.4	1.98 (m), 2.32 (m)	36.6	1.62 (m), 2.32 (m)
3^II^	73.8	4.01 (m)	73.8	3.98 (m)	70.7	3.85 (m)
4^II^	74.6	3.82 (m)	74.7	3.79 (m)	80.0	3.23 (m)
5^II^	67.5	4.23 (m)	67.4	4.11 (m)	69.8	3.64 (m)
6^II^	17.9	1.40 (d, *J* = 6.3)	17.8	1.40 (m)	18.7	1.33 (d, *J* = 7.5)
-OMe	55.4	3.40 (s)	55.3	3.41 (s)		
β-D-cym			β-D-cym		α-L-cym	
1^III^	99.5	4.95 (overlap)	99.3	5.05 (overlap)	99.4	4.98 (d, *J* = 2.5)
2^III^	36.4	1.86 (m), 2.41 (m)	36.3	1.86 (m), 2.41 (m)	32.3	1.82 (m), 2.05 (m)
3^III^	77.9	3.87 (m)	77.6	3.86 (m)	77.9	3.85 (m)
4^III^	82.4	3.44 (m)	82.3	3.42 (m)	73.4	3.62 (m)
5^III^	69.5	4.21 (m)	69.3	4.21 (m)	65.1	4.48 (m)
6^III^	18.7	1.25 (d, J = 6.1)	18.3	1.26 (m)	18.7	1.50 (d, *J* = 6.0)
-OMe	58.3	3.51 (s)	58.2	3.46 (s)	56.8	3.37 (s)
α-L-cym			α-L-cym		β-D-cym	
1^IV^	99.1	5.05 (overlap)	98.9	5.16 (m)	95.5	5.19 (d, J = 10.0)
2^IV^	32.2	1.89 (m), 2.33 (m)	32.1	1.89 (m), 2.33 (m)	36.6	3.37 (m)
3^IV^	76.5	3.70 (m)	76.1	3.69 (m)	77.8	3.41 (m)
4^IV^	73.3	3.59 (m)	73.2	3.61 (m)	82.4	3.31 (m)
5^IV^	66.4	4.55 (m)	66.3	4.54 (m)	69.5	3.56 (m)
6^IV^	18.5	1.37 (d, *J* = 6.3)	18.1	1.37 (m)	18.7	1.35 (d, *J* = 6.5)
-OMe	56.7	3.37 (s)	56.5	3.37 (m)	57.1	3.39 (s)
					α-L-cym	
1^V^					99.1	4.96 (d, *J* = 3.0)
2^V^					32.3	1.93 (m), 2.37 (m)
3^V^					76.6	3.72 (m)
4^V^					73.1	3.57 (m)
5^V^					66.4	4.53 (m)
6^V^					18.8	1.49 (d, *J* = 6.0)
-OMe					58.4	3.42 (s)

* Compound **1** and **3**: in C_5_D_5_N; compound **2**: in CD_3_OD; cym: cymaropyranosyl; digin: diginopyranosyl; glu: glucopyranosyl; digit: digitoxopyranosyl.

The ^1^H-NMR spectrum of **2** showed three Me groups at *δ*_H_ 2.02 (s), 1.54 (3H, d, *J* = 6.0 Hz) and 1.33 (s), three oxygenated CH groups at *δ*_H_ 3.81 (1H, m), 3.93 (1H, m) and 5.18 (1H, m), one olefinic proton at *δ*_H_ 5.31 (1H, br s), one cinnamoyl group at *δ*_H_ 6.80 (1H, d, *J* = 12.0 Hz), 7.96 (1H, d, *J* = 12.0 Hz), 7.62 (2H, m) and 7.35 (3H, m) which was supported by the ion fragment at *m/z* = 147 [C_9_H_7_O_2_]^+^ arising from the aglycone moiety. From ^1^H-NMR and ^13^C-NMR, one nicotinoyl group [*δ* (H) 9.59 (1H, d, *J* = 1.6 Hz), 8.79 (1H, dd, *J* = 5.6, 1.6 Hz), 8.40 (1H, d, *J* = 7.8 Hz) and 7.28 (1H, d, *J* = 7.2 Hz); *δ* (C) 164.6 (s), 153.7 (d), 151.6 (d), 137.4 (d), 127.1 (s) and 123.9 (d)] was also found in the aglycone moiety. The ^1^H-NMR data of the aglycone portion of **2** was compared with the data of gagaminine [6], showing that the major difference was the coupling constant of H-2′ and H-3′ was 12.0 Hz, so it could be deduced that the relative configuration at C-2′ and C-3′ was *cis*. Thus, the aglycone of compound **2** was proposed to be 2′,3′-*Z*-gagaminine. In the NOESY spectrum, NOE correlations between H-9 (*δ*_H_ 1.71, m) and H-12 (*δ*_H_ 5.18, m) gave evidence for a 12-O-cinnamoyl group that was *β*-linked. According to the literature [8], the stereochemistry of the C-14 hydroxyl group was assigned as *β*. The chemical shifts of C (13) and C (14) appear at *δ* 56.9 and 88.8, respectively. It could be deduced that the C/D ring junction was *cis* compared with the same carbons at *d* 41.6–42.7 and 58.7–59.2 for the *trans* form [8]. By comparing the spectroscopic data of the sugar moiety in **2** with those of **1**, compound **2** was seen to possess the same sugar substitution pattern as that of **1**. Thus, compound **2** was determined to be 2′,3′-*Z*-gagaminine 3-*O*-*α*-L-cymaropyranosyl-(1→4)-*β*-D-cymaropyranosyl-(1→4)-*α*-L-diginopyranosyl-(1→4)-*β*-D-cymaropyranoside, and named cyanoauriculoside G.

Compound **3** was isolated as a white amorphous powder, and showed positive reactions in the *Libermann-Buchard* and *Keller-Killiani* tests, indicating the presence of another steroidal skeleton with a 2-deoxysugar moiety [5]. The molecular formula was established as C_65_H_96_O_23_ according to the HRESIMS spectrum (*m/z* 1,267.6268 [M+Na]^+^, calcd. 1,267.6240). The ^13^C-NMR data of the aglycone portion of **3** were compared with those of kidjoranin [9], and showed that the major difference was the presence of an additional acetyl group [*δ* (H) 2.06 (3H, s); *δ* (C) 170.6 (s), 21.2 (q)] in the structure of **3**. Thus, the aglycone of compound **3** was proposed to be 17-*O*-acetylkidjoranin, which was supported by NOE correlations between H-21 (*δ*_H_ 2.49, s) and H-11′ (*δ*_H_ 2.06, s), between H-16 (*δ*_H_ 2.05, 3.24, m) and H-11′ (*δ*_H_ 2.06, s). The 17-*α* configuration was confirmed by the observation that the carbonyl carbon of the *α*-linked methyl ketone at C-17 appears at *δ* 210.2, compared with *δ* 216 ppm for the *β* configuration [10]. Thus, the 17-O-acetyl group was *β*-linked. In the NOESY spectrum, NOE correlations between H-9 (*δ*_H_ 1.72, m) and H-12 (*δ*_H_ 5.18, m) provided evidence for a *β*-linked 12-O-cinnamoyl group. According to the literature [8], the stereochemistry of the C-14 hydroxyl group was assigned as *β*. The chemical shifts of C (13) and C (14) appear at *δ* 58.2 and 89.6, respectively. It could be deduced that the C/D ring junction was *cis* [8]. With respect to the glycosidic portion, the ^13^C-NMR spectra showed five anomeric carbon signals at *δ*_C_ 96.4, 99.7, 99.4, 95.5 and 99.1, corresponding to anomeric H-atom signals at *δ* (H) 4.89 (d, *J* = 9.5 Hz), 5.12 (d, *J* = 10.0 Hz), 4.98 (d, *J* = 2.5 Hz), 5.19 (d, *J* = 10.0 Hz) and 4.96 (d, *J* = 3.0 Hz), which indicated that there were five sugar moieties in **3**. Acidic hydrolysis of **3** afforded a sugar mixture of cymarose and digitoxose, as determined by TLC comparison with authentic samples. The sugar moiety was linked to the C (3)-O of the aglycone, which was supported by HMBC correlations between H-C (1^I^) at *δ* (H) 4.89 and C (3) at *δ* (C) 77.7. Signals of each sugar unit (Table 2) were assigned by the HSQC, HMBC and ^1^H-^1^H COSY analyses and sugar moieties were identified as two *α*-L-cymaropyranosyls, one *β*-D-cymaropyranosyl and two *β*-D-digitoxopyranosyls. The sequence of the sugar chain was determined by HMBC spectrum, in which distinct correlations between H-C (1^II^) at *δ* (H) 5.12 and C (4^I^) at *δ* (C) 83.5, between H-C (1^III^) at *δ* (H) 4.98 and C (4^II^) at *δ* (C) 80.0, between H-C (1^IV^) at *δ* (H) 5.19 and C (4^III^) at *δ* (C) 73.4, and between H-C (1^V^) at *δ* (H) 4.96 and C (4^IV^) at *δ* (C) 82.4. Thus, compound **3** was determined to be 17-*O*-acetylkidjoranin-3-*O*-*α*-L-cymaropyranosyl-(1→4)-*β*-D-cymaropyranosyl-(1→4)-*α*-L-cymaropyranosyl-(1→4)-*β*-D-digitoxopyranosyl-(1→4)-*β*-D-digitoxopyranoside, named cyanoauriculoside H.

The known constituents were identified as gagaminine 3-*O*-*α*-L-cymaropyranosyl-(1→4)-*β*-D-cymaropyranosyl-(1→4)-*α*-L-diginopyranosyl-(1→4)-*β*-D-cymaropyranoside (**4**) [6], and wilfoside D1N (**5**) [11] by comparison of their spectroscopic data with those reported in the literature.

## 3. Experimental

### 3.1. General

Column chromatography was carried using silica gel (200–300 mesh), and Thin-Layer Chromatography (TLC) was performed on silica gel GF_254_ from the Qingdao Haiyang Chemical Group Co., P. R. China. RP-18 silica gel was purchased from YMC CO., LTD., Japan. NMR spectra were run on a Bruker DRX-500 MHz spectrometer with TMS as internal standard. HRESIMS were measured on Micromass Q-Tof-Ultima mass spectrometer. The optical rotation was measured on a Jasco P-1020 polarimeter. HPLC was performed on an Ultimate 3000 apparatus using 5C18-MS-II column (ODS, 250 × 10 mm, 5 μm) and monitored by an UV detector.

### 3.2. Plant material

The roots of the *C. auriculatum* were collected from Jishou, Hunan Province, P. R. China, in September 2007, and identified by Prof. Ding-Rong Wan. The voucher specimen (07091201) was deposited in the Herbarium of College of Pharmacy, South Central University for Nationalities.

### 3.3. Extraction and isolation procedures

The aerial roots of *C. auriculatum* (4 kg) were powdered and extracted three times with 95% EtOH at room temperature (48, 48 and 24 h, 6 L × 3). The ethanolic extract (0.6 kg) was suspended in water (1.6 L) and then successively partitioned with petroleum ether (1.5 L × 3), CHCl_3_ (1.5 L × 3), EtOAc (1.5 L × 3) and *n*-BuOH (1.5 L × 3). The CHCl_3_ extract (195 g) was chromatographed on the silica column using gradient solvents of cyclohexane/EtOAc (100:0→0:100) and EtOAc/MeOH (100:0→0:100) to yield seven fractions (fr.1-fr.7). Fr.2 (4.3 g) was repeatedly chromatographed over a silica gel, then purified on a RP-C_18_ silica gel column to afford **1** (27 mg) and **5** (28 mg). Fr.4 (3.0 g) was subjected to CC (ODS, H_2_O/MeOH 9:1→1:9 gradient system) to afford 4 subfractions (Fr.4.1-Fr.4.4). Fr.4.1 was purified by semi-prep. HPLC (MeOH/H_2_O 80:20, 3 mL/min, t_R_ 19.3 min) to yield **2** (30 mg). Fr.4.2 was purified by semi-prep. HPLC (MeOH/H_2_O 80:20, 3 mL/min, t_R_ 23.7 min) to yield **4** (26 mg). Fr.4.3 was purified by semi-prep. HPLC (MeOH/H_2_O 80:20, 3 mL/min, t_R_ 29.8 min) to yield **3** (32 mg). 

### 3.4. Acid hydrolysis

A soln. of **1**, **2**, and **3** (each 5 mg) in MeOH was treated with 0.05 mol/L HCl, 4-dioxane 1:1 (1 mL) at 60 °C for 1.5 h, respectively. After removing dioxane, the soln. was extracted with EtOAc (3 × 2 mL). The aq. layer was neutralized by NaOH and concentrated under reduced pressure to give the sugar fraction. The presence of the monosaccharides in the hydrolysates of each compound was confirmed by TLC comparison with authentic samples. Cymarose was detected from compounds **1**–**3**; diginose was detected from compounds **1** and **2**; digitoxose was detected from compound **3**. The R_f_ values of di gitoxose, diginose and cymarose were 0.51, 0.66 and 0.76, respectively with CHCl_3_: MeOH (95:5), 0.07, 0.18 and 0.23, respectively with P.E.: Me_2_CO (3:1).

### 3.5. Physical data of new compounds

*20-O-acetyl-8,14-seco-penupogenin-8-one 3-O-α-L-cymaropyranosyl-(1→4)-β-D-cymaropyranosyl-(1→4)-α-L-diginopyranosyl-(1→4)-β-D-cymaropyranoside* (**1**). White amorphous powder; UV λ_max_ (MeOH) nm (logε): 280 (4.35); [α]_D_^20^ = + 19.2 (c 0.20, MeOH); For ^1^H-NMR and ^13^C-NMR spectroscopic data (in C_5_D_5_N), see Table 1 and Table 2; HRESIMS [M+Cl]^-^*m/z* 1,165.5694 (calcd. for C_60_H_90_O_20_: 1,165.5713). 

*2′,3′-Z-gagaminine 3-O-α-L-cymaropyranosyl-(1→4)-β-D-cymaropyranosyl-(1→4)-α-L-diginopyranos-yl-(1→4)-β-D-cymaropyranoside* (**2**). White amorphous powder; UV λ_max_ (MeOH) nm (logε): 203 (4.32); [α]_D_^20^ = + 21.6 (c 0.22, MeOH); For ^1^H-NMR and ^13^C-NMR spectroscopic data (in CD_3_OD), see Table 1 and Table 2; HRESIMS [M+Na]^+^*m/z* 1,216.6033 (calcd. for C_64_H_91_NO_20_Na: 1,216.6032).

*17-O-acetyl-kidjoranin 3-O-α-L-cymaropyranosyl-(1→4)-β-D-cymaropyranosyl-(1→4)-α-L-cymaro- pyranosyl-(1→4)-β-D-digitoxopyranosyl-(1→4)-β-D-digitoxopyranoside* (**3**). White amorphous powder; UV λ_max_ (MeOH) nm (logε): 278 (4.37); [α]_D_^20^ = - 60.2 (c 0.25, MeOH); For ^1^H-NMR and ^13^C-NMR spectroscopic data (in C_5_D_5_N), see Table 1 and Table 2; HRESIMS [M+Na]^+^ m/z 1,267.6268 (calcd. for C_65_H_96_O_23_Na: 1,267.6240).

## 4. Conclusions

Twenty compounds were isolated from the dry roots *C.auriculatum Royle ex Wight*, including thirteen C-21 steroidal glycosides. The anti-tumour activity of these C-21 steroidal glycosides compounds has been studied. Further research on isolation and identification of more bioactive compounds will be helpful to understand this traditional medicine.
